# Role of sphingolipid metabolites in the homeostasis of steroid hormones and the maintenance of testicular functions

**DOI:** 10.3389/fendo.2023.1170023

**Published:** 2023-03-17

**Authors:** Defan Wang, Yedong Tang, Zhengchao Wang

**Affiliations:** ^1^ Fujian Provincial Key Laboratory of Reproductive Health Research, School of Medicine, Xiamen University, Xiamen, China; ^2^ Fujian Provincial Key Laboratory for Developmental Biology and Neurosciences, College of Life Sciences, Fujian Normal University, Fuzhou, China

**Keywords:** steroid hormone, gametogenesis, sphingosine-1-phosphate, sphingomyelin, testicular function

## Abstract

With the acceleration of life pace and the increase of work pressure, the problem of male infertility has become a social problem of general concern. Sphingolipids are important regulators of many cellular processes like cell differentiation and apoptosis, which are ubiquitously expressed in all mammalian cells. Various sphingolipid catabolic enzymes can generate multiple sphingolipids like sphingosine-1-phosphate and sphingomyelin. Present studies have already demonstrated the role of steroid hormones in the physiological processes of reproduction and development through hypothalamus-pituitary-gonad axis, while recent researches also found not only sphingolipids can modulate steroid hormone secretion, but also steroid hormones can control sphingolipid metabolites, indicating the role of sphingolipid metabolites in the homeostasis of steroid hormones. Furthermore, sphingolipid metabolites not only contribute to the regulation of gametogenesis, but also mediate damage-induced germ apoptosis, implying the role of sphingolipid metabolites in the maintenance of testicular functions. Together, sphingolipid metabolites are involved in impaired gonadal function and infertility in males, and further understanding of these bioactive sphingolipids will help us develop new therapeutics for male infertility in the future.

## 1 Introduction

Sphingolipids are a lipid family with a common sphingomyelin base skeleton, which participates in various physiopathological processes of cells, including steroid production, cell differentiation and apoptosis (1–12). In the mammalian reproductive system, many types of cells can produce sphingolipid metabolites, especially ceramide and sphingosine-1-phosphate, which are the second messengers (13–18). Therefore, sphingolipid metabolites in reproductive system have attracted much attention in recent years.

Steroid hormone is an important regulator of many physiological processes in mammalians (19–21). It is specifically synthesized in steroid-producing tissues through a series of multi-step reactions under the catalysis of monooxygenase and hydroxysteroid dehydrogenase superfamily members (20–23). It plays a very important regulatory role in maintaining testicular functions (19–27). Many sphingolipid metabolites can regulate the signaling pathways of steroid production, through acting as a second messenger in the signaling cascade to participate in the regulation of reproductive functions (5, 15, 17, 27–29).

Recent studies have shown that sphingolipid metabolites not only participate in the regulation of steroid biosynthesis (5, 16, 17, 21, 22, 24, 30), but also participate in the production of male sperm, and mediate the apoptosis of germ cells induced by stress or injury (9, 15, 27, 28), indicating that sphingolipids may be related to the impairment of gonadal function and infertility. In general, this research field provides an exciting direction for basic biology and clinical medicine, which can further study the possible role of abnormal sphingolipids in male infertility.

In the present review, we summarized the research progress on the role of sphingolipid metabolites in steroid hormone balance and testicular function maintenance during recent years, providing a theoretical basis to develop new therapies for male fertility.

## 2 Sphingolipid and its metabolites

Sphingolipids have always been considered as the structural inert component of the cell membrane with sphingomyelin base skeleton (31–33). Okazaki et al. found that in various types of cells, different stimuli can promote sphingomyelinase to split sphingomyelin, and then send signals (31, 34). Since sphingosine can inhibit PKC (35–37), people have generated great interest in sphingomyelin metabolites.

**Figure 1:**
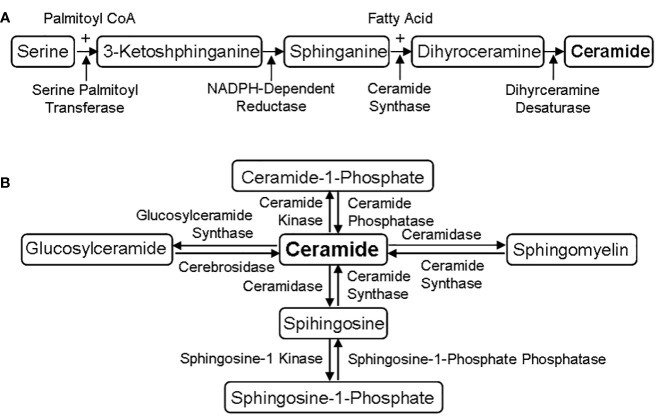
Sphingolipid metabolic pathways. **(A)** *De novo* synthesis of sphingolipids. The *de novo* biosynthesis of sphingolipids is from serine and palmitoyl CoA, and finally converted into ceramide through four-step reactions under the catalysis of serine palmitoyl transferase, NADPH-dependent reductase, ceramide synthase and dihyrceramine desaturase. **(B)** Different salvage of sphingolipids. Ceramide is the precursor of all sphingolipids, which can be converted into other phospholipids under the action of specific enzymes, such as ceramide synthase and ceramidase.

The biosynthesis of sphingolipids is from serine and palmitoyl CoA, and finally converted into ceramide, the precursor of all sphingolipids (33). The process of sphingolipid metabolism includes two stages, *de novo* synthesis (**Figure 1A**) and following salvage (**Figure 1B**). More and more evidences show that sphingolipids, as a structural component of cell membrane, have a signaling transduction function (11, 31, 32), and the special membrane micro-region rich in cholesterol and sphingolipid is considered as the center of signal molecular organization, participating in the signaling transduction of G protein-coupled receptor and growth factor receptor (9, 31, 32). Therefore, sphingolipids are widely considered to play an important role in cell signaling at present.

At present, a variety of sphingolipid metabolites have been found and their physiological effects have also been clarified, such as glycosphingolipids (31, 33, 38). These molecules are similar in structure and can be transformed into each other, but their biological functions are different (31, 38). Given the low concentration of these sphingolipid metabolites, they may function as an important signaling molecules participating in various cell events, like cell cycle arrest, apoptosis and intercellular interaction, such as S1P and C1P (31, 39). C1P is the main metabolite of ceramide, which can inhibit cell apoptosis and induce cell survival (18, 35, 39), besides prostaglandin synthesis and arachidonic acid release (5, 19, 27). Like ceramide, SPH participates in apoptosis (5, 15) and also inhibits PKC (37, 40) and PLD (41). Noteworthy, sphingolipid metabolites are being extensively studied as potential anticancer targets, since many intracellular and extracellular cytokine regulates the dynamic balance between sphingolipid metabolites through ceramidase, SK and S1P phosphatase (3, 18, 30, 31, 34).

## 3 Testicular synthesis of steroid hormones

**Figure 2:**
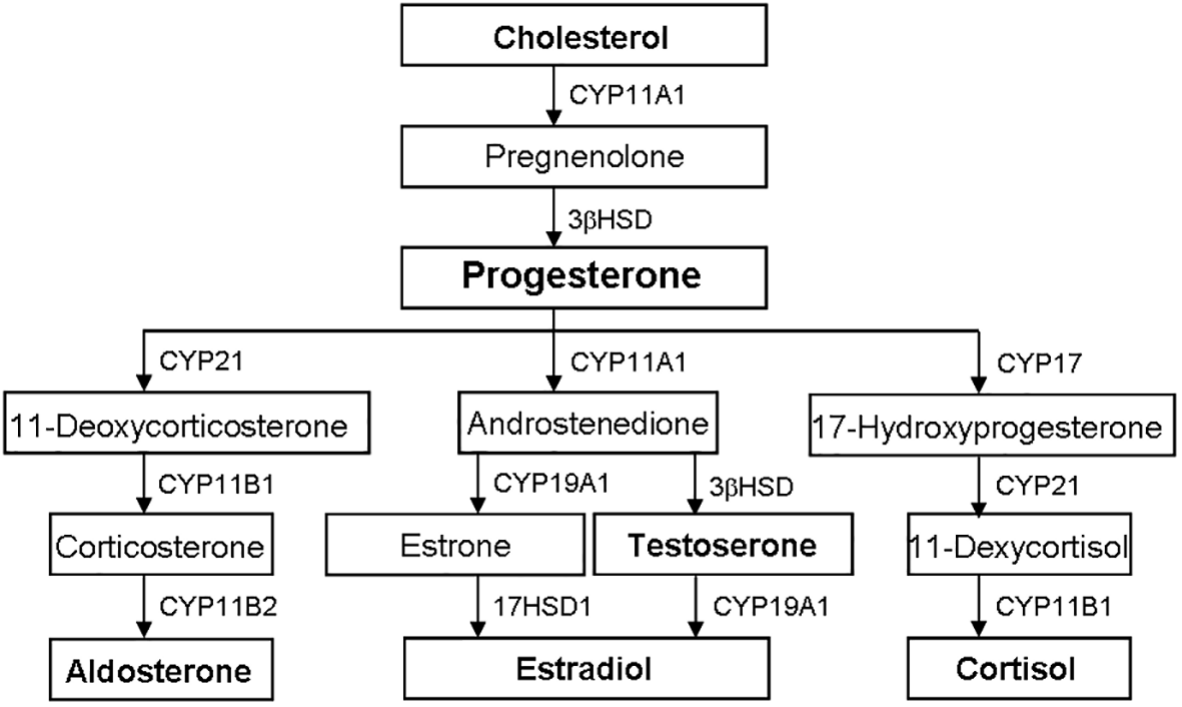
Biosynthetic pathways of steroid hormones. The biosynthesis of steroid hormones is from cholesterol through the sequence activity of CYP and HSD. Progesterone is the precursor of other steroid hormones, such as aldosterone, testosterone, estradiol and cortisol. CYP, cytochrome P450; HSD, hydroxysteroid dehydrogenase.

The biosynthesis of steroid hormones is from cholesterol through the sequence activity of CYP and HSD, mainly occurring in tissues such as testis, ovary, adrenal gland and placenta (**Figure 2**) (5, 15, 21, 27, 30, 38)

The main site of androgen biosynthesis is testis, and the expression of tissue-specific steroid gene is the reason for the difference of sex hormones (5, 17, 21). When the peptide hormone is combined with the homologous GPCR in the target tissue, the steroid biosynthesis is started, and then the steroid hormone is synthesized through the two different time stages of acute and chronic response stages, so as to ensure its secretion in an appropriate and controlled manner (5, 21, 30).

The acute response stage of steroid biosynthesis involves StAR activation. StAR can promote the rapid mobilization of cholesterol from the outer to the inner layer of mitochondrial membrane (42–44). Besides rate-limiting enzyme StAR, other proteins like PBR, HSL and SR-BI are also involved (42, 44–46).

The chronic response stage of steroid biosynthesis involves the transcription activation of steroid hormone genes. This process is mainly through activating various signaling like cAMP/PKA by adenylate cyclase for transcriptional activation (5, 21). SF-1 is one of the main transcription factors during this process, whose activity is regulated by phosphorylation and acetylation (38, 47, 48).

**Figure 3:**
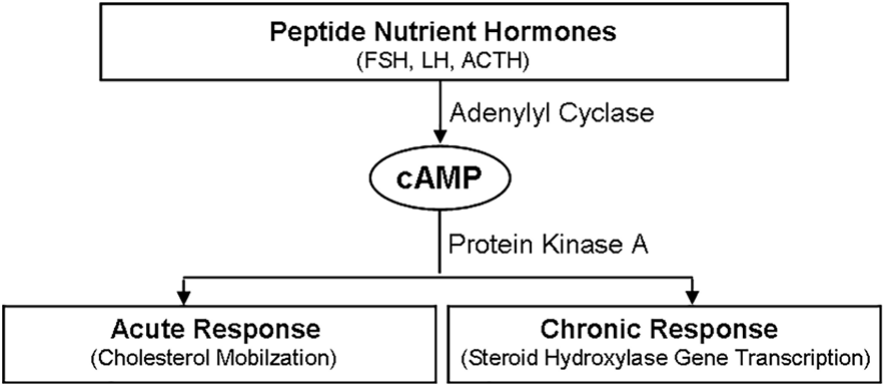
Regulatory Effects of peptide hormones on steroidogenesis. Peptide hormones, such FSH, LH and ACTH, initiate the synthesis of steroid hormones through cAMP/PKA signaling pathway, which includes two different time stages. One is the acute response stage, which is characterized by cholesterol mobilization. And the other is the chronic response stage, which is characterized by steroid hydroxylase gene transcription. FSH, follicle stimulating hormone; LH, luteinizing hormone; ACTH, adreno-cortico-tropic-hormone; PKA, protein kinase A; cAMP, cyclic adenosine monophosphate.

Many peptide hormones are involved in the initiation of steroidogenesis, such as FSH, LH and ACTH (16, 21). The combination of these peptide hormones to their homologous receptors leads to downstream signaling activation, mainly cAMP/PKA signaling, thus activating many target transcriptions (**Figure 3**) (5, 17). Besides cAMP/PKA, other signaling of cytokines and sphingolipids are also involved in regulating the synthesis of steroid hormones (5). The biosynthesis of testicular hormones is closely related to the specific expression profiles of steroid hormone synthases, such as CYP11A1 and 3βHSD, which are expressed in testis (21).

## 4 The role of sphingolipid metabolites in the synthesis of steroid hormones

**Figure 4:**
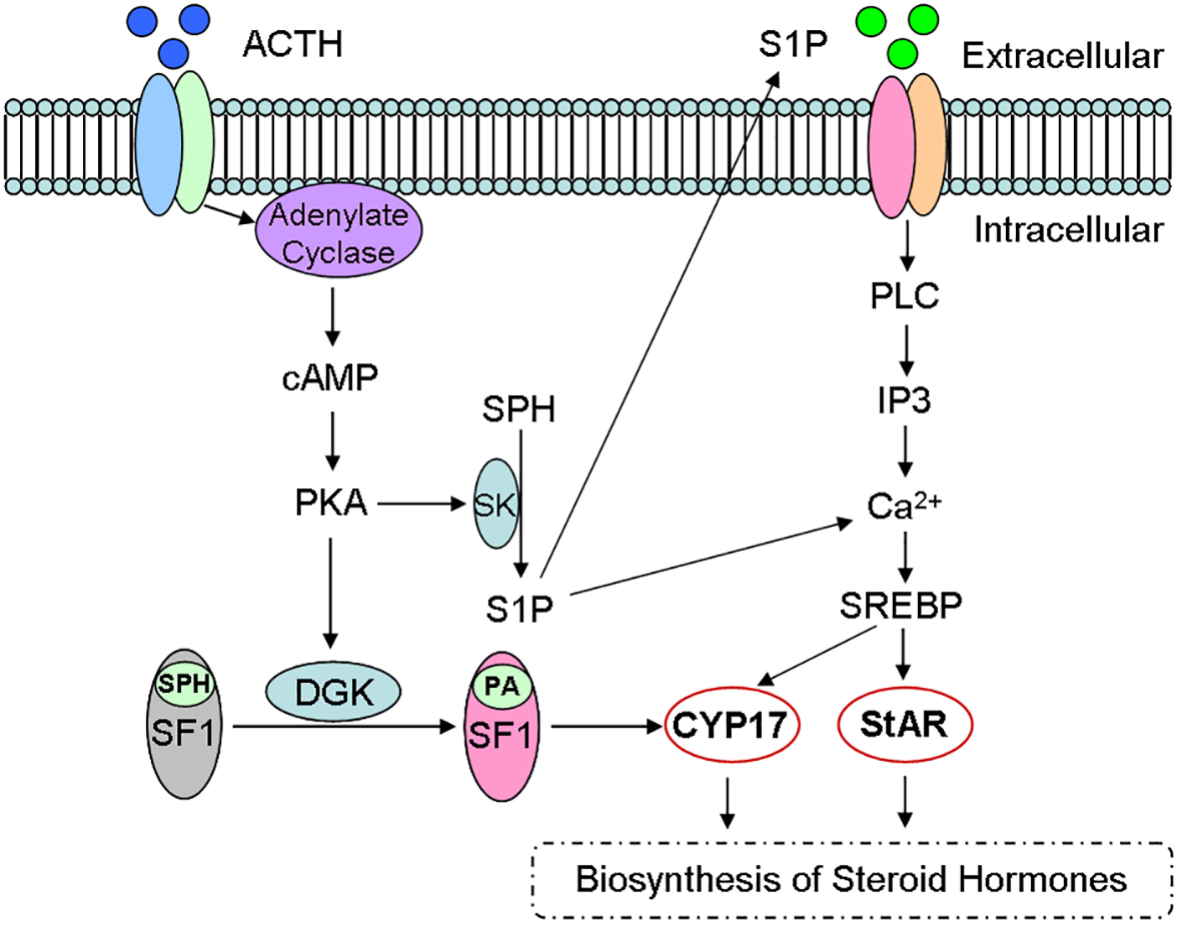
Sphingolipid metabolites and steroid biosynthesis. Steroid hormones are synthesized mainly *via* cAMP/PKA signaling pathway and sphingolipids act as the second regulator during the steroidogenesis. For example, ACTH activates cAMP/PKA signaling, reduces sphingomyelin number and increases S1P production, which participates in the biosynthesis of Steroid hormones through CYP and StAR activity. ACTH, adreno corticotropic hormone; cAMP, cyclic adenosine monophosphate; PKA, protein kinase A; DGK, diacylglycerol kinase; SPH, sphingosine; SF-1, steroid forming factor-1; PA, phosphatidic acid; SK, sphingosine kinase; S1P, sphingomyelin-1-phosphate; CYP, cytochrome P450; StAR, steroidogenic acute regulatory protein.

Although cAMP/PKA is main signaling, studies have found that sphingolipids can be used as the second regulator of steroid biosynthesis (**Figure 4**) (17, 21). For example, ACTH/cAMP can reduce the number of sphingomyelin, ceramide and SPH in the cells, and increase S1P production through SK activation (16). Bioactive sphingolipids can not only regulate the expression of CYP (5), but also act as the ligand of SF-1 (17, 47). Therefore, sphingolipids have been identified as one important synthesis regulator.

### 4.1 Ceramide and steroid biosynthesis

Ceramide can regulate cytokine-mediated signaling, like TNF-α, FasL, INF-γ and IL-1β, leading to the changes in steroid biosynthesis (17, 30, 49). They regulates ceramide concentration through activating intracellular sphingomyelinases (9, 38), but the mechanism of ceramide functions still remains to be further studied.

In Leydig cells, TNF-α and IL-1β can not only activate sphingomyelinases and generate ceramide (49), but also reduce StAR expression and testosterone secretion (50), while ceramide can inhibit testosterone synthesis induced by hCG (49).

Ceramide can also regulate the expression and activity of steroid synthases. For example, ceramide can regulate P450c17α expression and inhibit cAMP production (49). Ceramide can also induce C/EBPβ recruitment to 11β-HSD1 promoter, thus activating gene expression (51).

Lastly, ceramide metabolism enzymes also participate in steroid biosynthesis. For example, cortisol can increase sphingosine concentration through inducing N-acylsphingosine amidohydrolase 1 expression, leading to the inhibition of SF-1 functions during the regulation of steroid biosynthesis (17).

### 4.2 Sphingosine and steroid biosynthesis

Sphingosine is also confirmed as a SF-1 ligand, since SF-1 binds to phospholipids (17). Therefore, sphingosine is an antagonist of SF-1. Further investigation indicates that the combination of sphingosine with SF-1 leads to a decrease of CYP17 expression (17). In addition, phosphatidic acid is the endogenous ligand of SF-1 (52), since cAMP can promote phosphatidic acid binding to SF-1 and reverse sphingosine antagonism (**Figure 4**).

As SF-1 ligand, sphingosine adds another regulatory level of sphingomyelins during the synthesis of steroid hormones. Notably, sphingosine may regulate the process of development and sex differentiation through antagonizing SF-1, since SF-1 also regulates many related gene expressions during these processes (17, 27, 47).

### 4.3 Sphingosine-1-phosphate and steroid biosynthesis

S1P signaling is activated in and out of cells by binding to specific S1P receptors (52). There are five S1P receptors, which combines with multiple heterotrimeric G proteins to activate specific intracellular targets, such as PI-3K, ERK and Rho (10–12).

The binding of S1P and its homologous receptor is the main signaling transduction, and PI-3K/Akt and ERK pathways are its downstream targets (35, 37, 52). The relationship between PI-3K/ERK activation and steroid biosynthesis further indicates the important role of S1P during the regulation of steroid biosynthesis (**Figure 4**) (5, 16).

### 4.4 Sphingomyelin and steroid biosynthesis

Sphingomyelin exists in cell membrane, which is the target of many external signals, involving in the regulation of steroid biosynthesis (5, 32, 41). Sphingomyelin can be hydrolyzed into ceramide by sphingomyelinases (41), which is mediated by many stimulators, including TNF-α, IL-1β and FasL (39, 50). For example, S1P and C1P inhibit acid sphingomyelinase activity through the negative feedback loop of sphingomyelin metabolism pathway (52).

In Leydig cells, sphingomyelin hydrolysis is associated with cholesterol migration (21). In addition, lysosomal sphingomyelin can bind to SF-1 and cAMP can promote the separation of sphingomyelin from SF-1 (17, 47). Given the dynamic balance of different sphingolipid metabolites in specific cells, sphingomyelin play an important role in the regulation of steroid biosynthesis as a precursor of ceramide, lysosomal sphingomyelin and S1P (34).

### 4.5 Glycosphingolipids and steroid biosynthesis

Bioactive sphingomyelins participate in steroid biosynthesis, however whether steroid hormones are involved in sphingolipid metabolism is another issue, which is exemplified in the neurosteroidogenesis between sphingomyelin and isoproterenone (53, 54). Mellon et al. found that isoprenol treatment can reduce sphingomyelin and improve neurodegeneration (30, 55). At the same time, sphingomyelin is critical for testosterone synthesis (17, 27), and testosterone can regulate the level of renal sphingomyelin (43, 52). Although the exact mechanism of molecular regulation is still unclear, these demonstrate the importance of sphingolipid metabolites and the complexity of steroid biosynthesis.

## 5 The role of sphingolipid metabolites in spermatogenesis

In mammals, testes are mainly composed of two functional regions, the seminiferous tubules containing germ cells and sertoli cells, and the stroma containing Leydig cells (23, 26, 56). Among them, spermatogenesis is a complex physiological process, which mainly occurs in the seminiferous tubules and is regulated by the interaction between multiple hormones (FSH and LH) and cells (24, 46, 57). The seminal vesicle contains many hydrolases, including sphingomyelinase, which is involved in sperm phospholipid hydrolysis, and closely related to sperm capacitation and fertilization (27, 29, 38).

Glycosphingolipids are widely expressed in cell membrane (58) and involved in differentiation, growth and transmembrane signaling (4, 38). For example, glycosphingolipids GM1 distributed in spermatozoa (59), and GM3 distributed in sertoli cells of seminiferous tubules (60). Analysis of sphingomyelin acyl composition of in ram sperm found that VLC PUFAs only bind with sphingomyelin, but not with other phospholipids (28).

The study of fertile and sterile mice that block the biosynthesis of glycosphingolipids found that the synthesis of acidic subgroups of fucosylated glycosphingolipids was blocked in fertile and sterile mice, but the neutral subgroup of fucosylated glycosphingolipids was only missing in sterile mice (61). Glycosphingolipids not only play a role in the secretion of testosterone (62), but also is critical to the prolonged polarization of sperm and the stability of the sperm-sertoli cell contact (29).

At the initial stage of spermatogenesis, not only fucosylated glycosphingolipids, but also ceramide and sphingomyelin (VLC-PUFA contents) firstly appear in the pachytene spermatocyte stage (63), and ceramide and sphingomyelin also appears in spermatocytes 25-27 days after birth (64), these compounds are the main components of mammalian sperm head (65).

During the last step of sperm maturation, ceramide and sphingomyelin also play a very important role in the sperm capacitation (29), After sperm was treated with the agents of sperm capacitation, such as calcium, bicarbonate and albumin, sperm sphingomyelin was degraded to ceramide, which reduced the sphingomyelin/ceramide ratio several times (64). Therefore, the hydrolysis of sphingomyelin in sperm head may be the early biochemical change of sperm before fertilization (64), but the mechanism of its action in spermatogenesis and capacitation is still unclear and needs further research.

## 6 The role of sphingolipid metabolites in testicular cell apoptosis

Sphingolipid metabolites play not only an important role in spermatogenesis, but a regulatory role in apoptosis of germ cells after pathological stress of testis (19, 22, 23, 26, 27, 30).

Firstly, sphingomyelinase can inhibit gonadotropin-induced testosterone synthesis in Leydig cells, reduce the binding of gonadotropin to receptors, then decrease cAMP (17, 23, 27). In mammalian testis, Leydig cells can maintain spermatogenesis and androgen synthesis (17, 23, 30, 57), indicating that sphingomyelinase not only reduces androgen synthesis, but also leads to excessive apoptosis of spermatogonia and spermatocytes.

Secondly, ceramide is involved in the process of testicular cell apoptosis mediated by various pathophysiological stresses. When male rats were treated with toxic compounds, their testicular cells were mostly Fas-positive and more sensitive to FasL (66). During the spermatogenesis, FasL/Fad is involved in the loss of germ cells (66, 67) and FasL can induce the increase of intracellular ceramide level (68). In testicular cells, Fas can activate cell apoptosis through ceramide signaling (69), indicating that Fas pathway is a sensor for toxic substances to induce testicular injury.

Thirdly, S1P may also participate in the apoptosis of testicular cells, since S1P can effectively inhibit chemotherapy-induced apoptosis of ovarian cells. Suomalainen et al. examined the changes of intracellular sphingolipids and cell apoptosis markers, and then found the elevated ceramide contents during the early apoptosis following with activated caspase 3 and appeared DNA laddering (70). Interestingly, reactive oxygen species inhibitor (n-acetyl-L-cysteine), not ceramide synthetase inhibitor (fumonisin B1) suppressed testicular apoptosis without ceramide changes, while S1P can inhibit testicular apoptosis by 30% (70). Additionally, ceramide-initiated SAPK/JNK signaling and protein phosphatase 2A activation is required during stress-induced apoptosis, such as TNF-a, FasL and X-rays (69–74).

**Figure 5:**
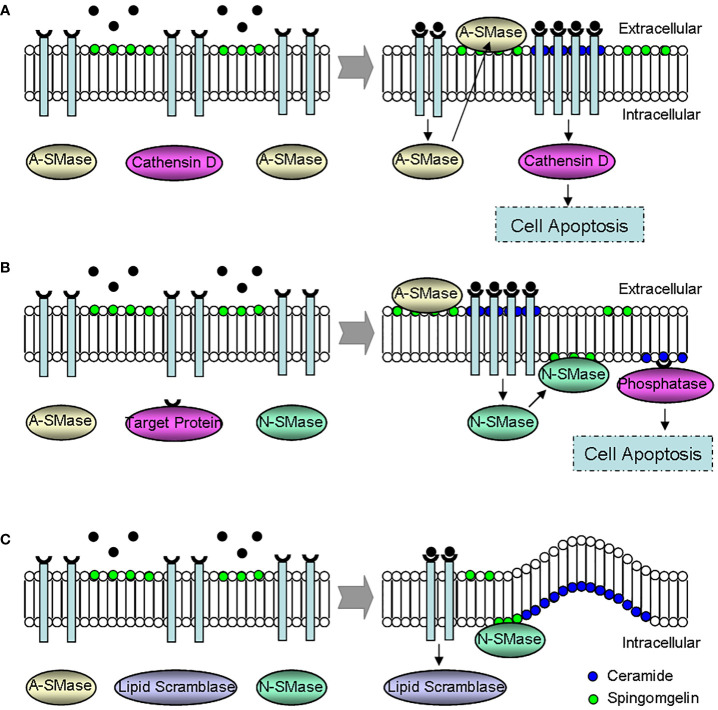
Effect mechanisms of ceramide during the regulation of cell apoptosis. **(A)** The instantaneous activation of A-SMase. A-SMase activation promotes ceramide-dependent receptor aggregation, leading to cell apoptosis *via* cathensin D. **(B)** The continuous activation of N-SMase. Ceramide induces cell apoptosis through the continuous activation of N-SMase and phosphatase. **(C)** The formation of membrane vesicles. The membrane morphology changes because of phospholipid disorder, and the membrane vesicle forms since N-SMase transforms sphingomyelin to excessive ceramide. N-SMase, neutral sphingomyelinase; A-SMase, acid sphingomyelinase.

Finally, ceramide is an important regulator of cell apoptosis (5, 9, 10, 18, 31, 35, 38, 61), which may play a role through the following three mechanisms (**Figure 5**). (i) Instantaneous acid sphingomyelinase activation increases the membrane ceramide, promotes receptor aggregation and subsequent apoptosis signaling transduction. (ii) Ceramide is continuously produced through the activation of neutral sphingomyelinase or ceramide activated protein phosphatase, resulting in cell apoptosis. And (iii) plasma membrane phospholipid disorder occurs, presenting sphingomyelin to neutral sphingomyelinase, producing ceramide, and then forming the morphological changes of membrane vesicles and apoptosis.

At present, the role of ceramide in testicular cell apoptosis has made great progress, but the role of other sphingolipid metabolites during the apoptotic process of testicular cells remains to be further clarified.

## 7 Summary and prospect

Together, the contribution of sphingolipid metabolites to the homeostasis of steroid hormones and the maintenance of testicular functions is reviewed, but it is necessary to fully clarify their roles in male reproduction in the future. These bioactive sphingolipids, as key regulators, regulate steroid biosynthesis at different levels, including the expression and activity of steroid synthetases. In addition, sphingolipid metabolites participate in the regulation of male gametogenesis and testicular damage. However, the mechanism of these active molecules (such as ceramide and S1P) during male gonadal development and gametogenesis needs further study, and mass spectrometry, metabolomic analysis and proteomics are important tools to further study the physiological and pathological functions of sphingolipid metabolites. The present review about sphingolipid metabolites will help to formulate new strategies to improve gamete maturation and survival rate, thus optimizing various assisted reproductive technologies. In addition, the selective regulation of these active molecules in germ cells will help to protect the testis from chemotherapy damage, preserve the fertility of prognosis and improve the quality of life.

## Author contributions

DW collected the literature and prepared the manuscript. YT collected the literature and made the illustrations. ZW conceptualized the article and revised the manuscript. All authors contributed to the article and approved the submitted version.
